# The Itch

**Published:** 1871-04

**Authors:** 


### THE ITCH.

It is worth having the itch to enjoy the bliss of scratching; there is nothing like it: why? You feel as if you could dig your nails an inch deep into the flesh. If, however, you get tired of scratching, take a grease, and you will soon be well. The books tell us that the common itch is occasioned by the bite of a living thing, so small, that it takes a hundred microscopes to see one of them. But the peculiarity of these little rascals is, that they are full of noses. A man has only one nose; and if you plug it up with putty, he will die in five minutes, if you can only induce him to keep his mouth shut; if he don't, putty it too. So as to the itch insect, you have only to putty him up; if, however, you are in a hurry to get well, keep the itching parts covered with sweet oil, and every one of them will be smothered to death.

